# Clonal Hematopoiesis after Autologous Stem Cell Transplantation Does Not Confer Adverse Prognosis in Patients with AML

**DOI:** 10.3390/cancers13133190

**Published:** 2021-06-25

**Authors:** Alexander D. Heini, Naomi Porret, Reinhard Zenhaeusern, Annette Winkler, Ulrike Bacher, Thomas Pabst

**Affiliations:** 1 Department of Medical Oncology, University Hospital and University of Bern, Center for Hemato-Oncology, University Cancer Center, 3010 Berne, Switzerland; alexander.heini@insel.ch; 2 Department of Hematology and Central Hematology Laboratory, Inselspital, Bern University Hospital, University of Bern, 3010 Berne, Switzerland; NaomiAzur.Porret@insel.ch (N.P.); veraulrike.bacher@insel.ch (U.B.); 3 Department of Medical Oncology, Spitalzentrum Oberwallis, 3900 Brig, Switzerland; reinhard.zenhaeusern@hopitalvs.ch; 4 Regionalspital Biel, 2501 Biel, Switzerland; anette.winkler@szb-chb.ch

**Keywords:** acute myeloid leukemia (AML), autologous stem cell transplantation (ASCT), clonal hematopoiesis (CH), prognosis, outcome

## Abstract

**Simple Summary:**

Around 50% of patients with acute myeloid leukemia (AML) achieve a definite cure with intensive chemotherapy and consolidation, but relapse remains the main cause of death. Clonal hematopoiesis (CH) describes the presence of a clonal subset of myeloid precursors without known hematologic disease. We aimed to investigate whether the presence of CH-related mutations in the three most common genes (*DNMT3A*, *TET2*, and *ASXL1*, called DTA mutations) after autologous stem cell transplantation (ASCT) influence the outcome and retrospectively analyzed samples of 110 AML patients. We found no significant impact from the presence of DTA-CH on progression-free or overall survival. Thus, the persistence of DTA mutations after induction treatment should not prevent AML patients in first remission from ASCT consolidation. These results should undergo verification in independent cohorts.

**Abstract:**

Introduction: Despite a 50% cure rate, relapse remains the main cause of death in patients with acute myeloid leukemia (AML) consolidated with autologous stem cell transplantation (ASCT) in first remission (CR1). Clonal hematopoiesis of indeterminate potential (CH) increases the risk for hematological and cardiovascular disorders and death. The impact of CH persisting after ASCT in AML patients is unclear. Materials and Methods: We retrospectively investigated the prognostic value of persisting *DNMT3A*, *TET2*, or *ASXL1* (DTA) mutations after ASCT. Patients underwent stratification depending on the presence of DTA mutations. Results: We investigated 110 consecutive AML patients receiving ASCT in CR1 after two induction cycles at our center between 2007 and 2020. CH-related mutations were present in 31 patients (28.2%) after ASCT. The baseline characteristics were similar between patients with or without persisting DTA mutations after ASCT. The median progression free survival was 26.9 months in patients without DTA mutations and 16.7 months in patients with DTA mutations (HR 0.75 (0.42–1.33), *p* = 0.287), and the median overall survival was 80.9 and 54.4 months (HR 0.79 (0.41–1.51), *p* = 0.440), respectively. Conclusion: We suggest that DTA-CH after ASCT is not associated with an increased risk of relapse or death. The persistence of DTA mutations after induction should not prevent AML patients in CR1 from ASCT consolidation. Independent studies should confirm these data.

## 1. Introduction

Patients with acute myeloid leukemia (AML) considered fit for intensive treatment are treated with one or two cycles of intensive induction chemotherapy, followed by consolidation with either additional chemotherapy cycles along with autologous or allogeneic hematopoietic stem cell transplantation. The stratification of patients for these consolidation treatment options relies on the genetic risk assessed by cytogenetics and molecular analyses at initial diagnosis, the response to induction treatment, and the morbidity and mortality associated with the treatment options [1,2]. The concept of minimal or measurable residual disease (MRD) after induction has a significant impact on the type of consolidation and, increasingly, maintenance treatment [3]. MRD is defined as the detection of malignant cells at a submicroscopic level, and it has been shown to be an independent prognostic factor for the risk of relapse and death [4]. Multiparameter flow cytometry as well as molecular methods are routinely used for diagnosis and screening for residual disease in AML patients. While recurrent mutations in genes such as *FLT3* or *NPM1* have been incorporated in risk assessment for a long time [1,3], the introduction of next-generation sequencing (NGS) into the hematologic routine has enabled the discovery of a multitude of additional and often novel mutations [5]. The detection of druggable mutations has also opened the door for customized treatment strategies, some of which were integrated in the European LeukemiaNet (ELN) risk assessment and treatment for patients with AML [1,6,7].

With increasing age, somatic mutations accumulate, owing to radiation, smoking, drug exposition, or impaired DNA repair mechanisms. It is estimated that around 10–15% of individuals aged around 70 and 20% of those aged around 90 have clonal hematopoiesis, which accounts for ≥10% of circulating nucleated cells [8]. This clonal hematopoiesis (CH) is defined as the presence of a somatic mutation associated with hematological malignancies such as *DNMT3A*, *TET2*, or *ASXL1* (DTA mutations) without morphologic or clinical evidence of a hematological neoplasm, similar to the monoclonal gammopathy of undetermined significance (MGUS) [9] or monoclonal B-cell lymphocytosis (MBL). The odds of progression of CH to an overt myeloid neoplasia are 0.5–1% per year [10]. Importantly, patients with CH were identified to be at a higher risk for the development of not only hematological malignancies, but also inflammation or vascular diseases and adverse survival outcomes [11,12].

Up to 50% of AML patients consolidated, after intensive chemotherapy induction, in first remission with high-dose chemotherapy and autologous stem cell transplantation (ASCT) achieve a definitive cure. Despite this, relapse remains the major cause of mortality in these patients, and outcomes vary widely between different subsets of patients [13,14,15]. While the presence of recurrent leukemia-associated mutations was shown to have an adverse prognostic impact in patients with AML undergoing intensive treatment, this correlation has yet to be explored for CH related mutations. Considering these facts, a more in-depth analysis assessing the role of CH in AML patients after autologous transplantation is still missing and warranted.

## 2. Materials and Methods

### 2.1. Study Design and Patients

This retrospective single-center analysis included consecutive patients with AML undergoing high-dose chemotherapy (HDCT) with autologous stem cell transplantation (ASCT) between February 2007 and February 2020 at the University Hospital of Bern in Switzerland. De novo and secondary AML cases were included. Data were collected for demographic details, peripheral blood values, bone marrow and peripheral blood cytomorphology, molecular and cytogenetic abnormalities, and marrow and blood assessments during the follow-up while under and following therapy. The primary study end points were progression-free and overall survival.

### 2.2. Treatment

Patients were treated within or according to the following protocols of the HOVON (a Dutch-Belgian hemato-oncology group) and the Swiss Group for Clinical Cancer Research (SAKK) AML groups: SAKK/HOVON-42, -81, -92, -102, and -132. Induction treatment consisted of one or two cycles of cytarabine and an anthracycline (either idarubicin or daunorubicin). Consolidation treatment consisted of HDCT with melphalan and cyclophosphamide or treosulfan, followed by autologous stem cell transplantation. Patients had to have an Eastern Cooperative Oncology Group performance status of 0–2 and no previous treatment for AML, with the exception of hydroxyurea for initial cytoreduction. The stem cells were harvested from peripheral blood after G-CSF stimulation in all cases.

### 2.3. Risk and Response Assessment

Genetic and molecular biologic risk profiling were applied according to the European LeukemiaNet (ELN) update from 2017 [1]. The response was assessed according to international working group (ELN) criteria. Morphologic complete remission (CR) was defined as bone marrow blasts of <5% with complete hematologic recovery (absolute neutrophil count (ANC) ≥ 1.0 G/L, platelets ≥ 100 G/L, and no transfusion dependence) [16].

### 2.4. Survival

Progression-free survival (PFS) was defined as the time from ASCT to disease progression or death from any cause or the last follow-up, whichever occurred first. Overall survival (OS) was defined as the time between ASCT and death from any cause or until the last follow-up. Subjects lost to the follow-up were censored at the time last known to be alive.

### 2.5. Molecular Analyses

Molecular analyses were performed during first complete remission as described above. Samples were taken 113 days after ASCT in the median (93 days, ranging from 154 days before to 5793 days after ASCT in the CH group and 134 days, ranging from 181 days before to 3524 days after ASCT in the no CH group). For patients lost to the follow-up or death, analyses were performed from cryoconserved material stored upon ASCT. Of the samples, 60.0% were derived from peripheral blood, 37.2% were from bone marrow, and 2.8% were analyzed in the stem cell product after apheresis. Next-generation sequencing (NGS) was performed by a ThermoFisher Ion Torrent S5. Analyses included *ASXL1* exons 11–12 (fragment length analysis for exon 12), *DNMT3A* exons 11–23, and *TET2* (whole gene).

### 2.6. Assessment and Definition of Clonal Hematopoiesis

Molecular mutation assessments were performed at the initial diagnosis and during a follow-up. CH was defined as the persistence of mutations in *DNMT3A*, *TET2*, or *ASXL1* at a variant allele frequency (VAF) level of at least 2% during complete remission [8]. Different cutoffs (5% and 10%) were also tested. DTA mutations that were cleared after intensive chemotherapy and ASCT were considered leukemia-related, whereas persisting DTA mutations with VAF levels of ≥2%, despite achieving hematologic CR and despite of the clearance of other coincidental mutations at a follow-up, were considered to be CH.

### 2.7. Statistical Analyses

All reported *p*-values were from two-tailed Fisher’s or unpaired *t*-tests. A value of *p* < 0.05 was considered statistically significant. Survival analyses were performed using the log-rank method. Analyses for the median follow-up were performed using reverse Kaplan–Meier estimates; thus, no *p* value was reported. Statistical analyses were performed in GraphPad Prism Version 8 (GraphPad Software, Inc., LaJolla, CA, USA).

## 3. Results

### 3.1. Patients

We identified 110 consecutive patients with newly diagnosed AML undergoing ASCT in first remission after intensive induction chemotherapy. The clinical characteristics of all patients are shown in Table 1. The median age was 54 years in both groups (*p* = 0.415), and 54.8% of patients in the CH group and 44.3% in the no CH group were female (*p* = 0.397). Favorable risk disease, according to ELN guidelines, was present in 45.2% of the patients in the CH group and 50.6% of those in the non-CH group (*p* = 0.674), while 48.4% versus 41.7% (*p* = 0.670) had intermediate risk AML, and 6.5% versus 7.6% (*p* > 0.999) had adverse risk. The subgroups were equally distributed with the exception of patients with both mutated *NPM1* and *FLT3*-ITD, which were more common in the CH group (19.4 vs. 5.1%, *p* = 0.029).

### 3.2. Treatment

The patients were treated within the previously reported HOVON/SAKK protocols. Induction treatment consisted of standard 7 + 3 chemotherapy, given for two cycles. The median time from diagnosis to ASCT was 3.4 months in the CH group and 3.7 months in the group without CH (*p* = 0.153). Conditioning for ASCT consisted of *BuCy* (1 mg/kg q6h oral busulfan on days -5 to -2 for a total dose of 16 mg/kg and 60 mg/kg i.v. cyclophosphamide on days -2 and -1) in 25 (80.7%) and 63 (79.6%) patients, *BuMel* (1mg/kg q6h oral busulfan on days -;5 to -2 and 140 mg/m^2^ i.v. melphalan on days -2 and -1) in 4 (12.9%) and 9 (11.4%) patients or with *TreoMel* (14 mg/m^2^ i.v. treosulfan on days -4 to -2 and 140 mg/m^2^ i.v. melphalan on day -1) in 2 (6.5%) and 7 (8.9%) patients (Table 1). The stem cells were retransfused on day 0. The number of re-transfused CD34+ cells was not different between patients with CH and without CH (3.8 and 4.2 × 10^6^ cells/kg body weight, *p* = 0.448). Of the patients with *IDH2* mutations, two without CH (2.5%) and one patient (3.2%) with CH underwent maintenance therapy with enasidenib after ASCT, while one patient with CH and *FLT3*-ITD received midostaurin maintenance, and 8 patients with adverse risk cytogenetics and good response to induction treatment underwent autologous transplantation in the absence of an available donor or because the patients declined allogeneic transplantation. 

### 3.3. Clonal Hematopoiesis

Following the definition above, CH of DTA after autologous transplantation was observed in 31 patients (28.2%). The distribution of mutations at diagnosis and remission, as well as the variant allele frequencies of DTA mutations during remission, is summarized in Supplemental Figure S1. Mutations in *DNMT3A* were the most common and were observed in 24 patients (77.4%), whereas *TET2*-CH mutation was found in 7 patients (22.6%) and *ASXL1*-CH mutation was found in 4 patients (12.9%). Meanwhile, four patients (12.9%) had DTA-related CH mutations in more than one gene (3 *DNMT3A* + *TET2*, 1 *DNMT3A* + *ASXL1*), while two patients with CH had two mutations in the same gene (1 *DNMT3A*, *1 TET2*).

Leukemia-related DTA mutations with molecular clearance after leukemia treatment were present in an additional 13 patients (11.8%). Again, the distribution was similar to the patients with CH-related mutations, with *DNMT3A* being most frequent (six patients (46.2%)) followed by *TET2* (five patients (38.5%)) and *ASXL1* mutations (two patients (15.4%)).

### 3.4. Survival Analysis

**Figure 1:**
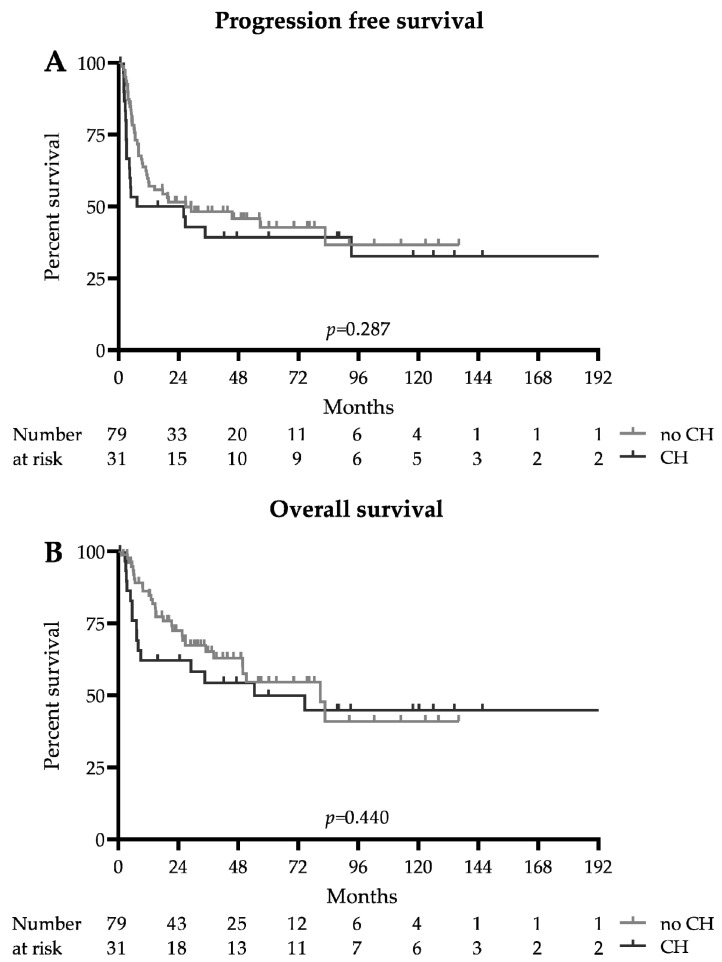
Survival: Kaplan–Meier survival estimates of patients with and without DTA clonal hematopoiesis (CH). (**A**) Progression-free survival. (**B**) Overall survival.

The survival data are summarized in Figure 1 and Table 2. The median follow-up for the whole cohort was 51.3 months (93 months in the CH group and 46.1 months in the no CH group). The median progression-free survival was 26.9 months in patients without DTA mutations and 16.7 months in patients with CH-related DTA mutations (HR 0.75 (0.42–1.33), *p* = 0.287). The median overall survival was 80.9 and 54.4 months, respectively (HR 0.79 (0.41–1.51), *p* = 0.440). Early mortality at 100 days after ASCT was higher in the CH group (12.9 vs. 1.3%, *p* = 0.022); however, this did not translate into adverse long-term survival, as the 2-year progression-free survival probability was 0.39 (0.34–0.57) in the CH group and 0.46 ((0.22–0.56), *p* = 0.391) in the no CH group, and the 2-year overall survival probabilities were 0.54 (0.35–0.71) and 0.63 ((0.49–0.73), *p* = 0.251), respectively. The relapse rate was similar between the CH and no CH group (51.6 vs. 41.7%, *p* = 0.398), and the number of patients undergoing allogeneic hematopoietic stem cell transplantation in CR2 did not differ significantly (19.4 vs. 24.1%, *p* = 0.801).

### 3.5. Survival of DTA Subgroups

Survival analysis of the DTA subgroups is summarized in Supplemental Figure S2. When analyzing the *DNMT3A*, *TET2*, and *ASXL1* CH-mutations individually, patients with persistent mutations in *TET2* and *DNMT3A* mutations had similar survival outcomes to patients without CH, while patients with persistent *ASXL1* mutations showed a non-significant trend toward better outcomes than patients without CH in the respective gene. However, the small number of patients in the *TET2* and *ASXL1* subgroups did not warrant a conclusive statement.

### 3.6. Secondary Malignancies

Three patients—two in the no CH group and one in the CH group—developed a second malignancy after ASCT. One patient was diagnosed with extensive disease small-cell lung cancer 6 years after ASCT, and one was diagnosed with metastatic adenocarcinoma of a gastrointestinal origin 7 years after ASCT. Both patients succumbed to these diseases. Most interestingly, one patient who underwent ASCT for AML with mutated *NPM1* and *FLT3*-ITD and who had no DTA-CH was diagnosed with AML with an *IDH2* mutation 8.5 years later without evidence of either mutated *NMP1* or *FLT3*-ITD or DTA mutations.

### 3.7. Different VAF Cutoffs

For the principal analysis, we applied a VAF cutoff for the definition of CH of 2% [8]. We also investigated the impact on the survival rates by applying 5% and 10% VAF cutoffs for the CH DTA mutations. Elevation of the VAF cutoff had no significant impact on survival, as summarized in Supplemental Figure S3.

## 4. Discussion

The increasing availability of NGS has led to the discovery of a multitude of novel mutations of, in part, indeterminate significance in AML, as well as other malignancies [9,17]. The prevalence of CH-related DTA mutations is known to increase with age, and patients with CH have an increased risk of developing hematologic malignancies, cardiovascular disorders and death [8]. Previous investigations found evidence of CH in between 65% and 75% of patients with AML [18,19,20]. This rate is higher than that in our cohort; however, the mentioned studies investigated more genes and used VAF cutoffs between 0.5% and 1%.

While the impact of CH on the risk of disease development has been intensively studied, less data are available on the impact of DTA mutations during complete remission and, to the best of our knowledge, no previous study has investigated the prognostic impact of CH for patients undergoing HDCT or ASCT for AML in CR1.

Autologous stem cell transplantation is one possibility for consolidation treatment for patients with good and intermediate risk AML in first complete remission. In ASCT, cells are harvested after induction and reinfused after high-dose chemotherapy consolidation to shorten marrow aplasia and reduce the risk for infectious complications. Harvesting stem cells from patients with hematologic malignancies poses a potential risk for the retransfusion of mutant cells. Retransfusion of DTA mutant cells may therefore pose a risk for development of a hematologic malignancy, AML relapse, or death.

First, we observed CH for DTA mutations at a frequency of 28.2% in our cohort during complete remission, which was similar to previous analyses in AML patients. Jongen-Lavrencic et al. reported a DTA-CH rate of 23% during complete remission, while Grimm et al. reported CH-related DTA mutations to be present at a rate of 35.4% during CR [4,21]. The detected rate of CH was also similar to that reported by Mouhieddine et al. in patients with multiple myeloma (21.6%) [22] or by Gibson et al. in patients with lymphoma (29.9%) [23] prior to ASCT. However, these analyses included small numbers of other CH-related mutations such as *TP53* or *PPM1D*.

Secondly, our analysis found no evidence for more adverse progression-free or overall survival outcomes in patients with persistent DTA-CH after HDCT or ASCT compared with patients without this phenomenon. These results are in line with a previous study by Jongen-Lavrencic et al. that showed no correlation between the detection of CH-related DTA mutations during complete remission and adverse outcomes in AML patients [4]. This analysis included a limited number of 78 patients (18%) that underwent ASCT, whereas almost half of the patients (44%) underwent allogeneic transplantation as a consolidation treatment. In comparison, our study cohort consisted of a homogenous and rather large sample of 110 mostly good and intermediate risk patients undergoing HDCT or ASCT as a consolidation in first remission.

Early mortality in the first 100 days was higher in the no CH group, but the reason for this finding remains unclear and, importantly, this did not translate into adverse long-term outcomes. Larger cohorts are needed to exclude any by chance findings. Similarly, the number of patients dying due to non-AML related reasons remained too small to allow or support conclusions on the development of or death from, for example, vascular disease.

In light of our results, the presence of DTA-CH in AML patients after HDCT or ASCT does not warrant intensification of treatment by allogeneic transplantation. Moreover, the presence of DTA-CH should not prevent eligible patients from undergoing autologous transplantation in first complete remission. While there was a tendency toward more adverse survival outcomes in patients with DTA mutations at VAFs over 10%, this did not reach statistical significance. Still, these patients may reflect a subset prone to relapse and death which may benefit from intensified surveillance and early intervention in the case of indication of a relapse.

In 2020, Gibson et al. investigated whether CH affecting allogeneic donors had an impact on the outcomes of 1727 patients receiving allogeneic HSCT from donors aged 40 years and older due to myeloid (53.8%) or lymphatic malignancies (41.6%). CH was present in a relevant proportion of 22.5% of donor samples. Surprisingly, evidence of donor *DNMT3A*-CH was associated with improved PFS and OS in the allo-transplant recipients, while other CH-related mutations (*TET2*, *ASXL1*, and *PPM1D*) had no impact on the outcomes. While the exact mechanism is unclear, it is suggested that *DNMT3A* mutations lead to improved T-cell immune activity, which is a key determinant of transplant activity. This is also supported by the observation that in patients who received post-transplant cyclophosphamide for GvHD prophylaxis, no impact due to the *DNMT3A* status on the survival outcome was observed. In addition, CH involving other gene mutations had no impact on the outcome [24]. Shlush et al. demonstrated that *DNMT3A* mutations can also be present in non-myeloid cells [25]. In our analysis, we found no difference in outcomes in the *DNMT3A*-CH subgroup compared with patients without CH in the respective gene. However, there were major differences in the patient population as well as treatment between our study and the mentioned study. In contrast, two investigations found the presence of CH involving *DNMT3A*, *TET2*, *ASXL1*, *TP53*, and *PPM1D* among others to be associated with inferior outcomes in patients undergoing autologous hematopoietic transplantation for multiple myeloma [22] and lymphoma [23]. Thus, possible differences in the long-term outcomes should be discussed in patients with CH receiving ASCT and in patients receiving allogeneic HSCT from donors with CH for different indications or different hematologic entities, respectively.

## 5. Conclusions

In conclusion, our study found no evidence of a worse prognosis for AML patients with DTA CH undergoing HDCT or ASCT in CR1. These data have a clinical impact, as they give no evidence for excluding AML patients with evidence of DTA CH from ASCT concepts. Our data should be independently confirmed in adequately powered prospective future studies. Due to the relatively small rate of progression from CH to overt hematologic malignancy (0.5–1% per year) [8,10], analyses from large registries with a sufficiently long follow-up will be needed to ultimately answer the issues raised in this study.

## Figures and Tables

**Table 1:** Baseline characteristics for patients included in the study. The *p* values are for differences between the DTA-CH (clonal hematopoiesis) and no CH groups. IQR: interquartile range; NOS: not otherwise specified; Mut: mutated; w/o: without; ASCT: autologous hematopoietic stem cell transplantation; BW: body weight. FLT3-ITD allelic ratios were <0.5 (low) and ≥0.5 (high).

Parameter	DTA-CH (*n* = 31)	No CH (*n* = 79)	Total (*n* = 110)	*p*
**Demographics**				
Female sex—no. (%)	17 (55)	45 (44)	52 (47)	0.397
Median age—years (IQR)	54 (41–63)	54 (40–61)	54 (40–61)	0.415
**Parameters at Diagnosis, Median (Range)**				
Leukocytes (G/L)	16.2 (1.2–303)	7.0 (0.6–240)	8.6 (0.6–303)	0.262
Neutrophils (G/L)	1.7 (0.2–50.2)	1.1 (0.0–19.4)	1.4 (0.0–50.2)	0.035
Platelets (G/L)	80 (9–268)	63 (7–714)	69 (7–714)	0.586
Hemoglobin (g/L)	99 (61–137)	89 (37–151)	95 (37–151)	0.163
Peripheral blasts (%)	44 (0–94)	41 (0–97)	44 (0–97)	0.924
Bone marrow blasts (%)	75 (30–95)	80 (0–95)	80 (0–95)	0.646
**ELN Risk Groups—no. (%)**				
Favorable	14 (45)	40 (51)	54 (49)	0.674
t(8;21)/*RUNX1-RUNX1T1*	2 (7)	9 (11)	11 (10)	0.725
inv(16)/*CBFB-MYH11*	2 (7)	8 (10)	10 (9)	0.722
Mut. *CEBPA*	1 (3)	4 (5)	5 (5)	>0.999
Mut. *NPM1* w/o *FLT3*-ITD/*FLT3*-ITD (low allelic ratio)	9 (29)	19 (24)	28 (26)	0.630
Intermediate	15 (48)	33 (42)	52 (43)	0.670
Mut. *NPM1* and *FLT3*-ITD (high allelic ratio)	6 (19)	4 (5)	10 (9)	0.029
NOS	9 (29)	29 (37)	38 (35)	0.509
Adverse	2 (7)	6 (8)	8 (7)	>0.999
Wild-type *NPM1* and *FLT3*-ITD (high allelic ratio)	2 (7)	1 (1)	3 (3)	0.191
Monosomal or complex karyotype	0	2 (3)	2 (2)	>0.999
t(v;11)/*KMT2A* rearranged	0	1 (1)	1 (1)	>0.999
Mut. *RUNX1*	0	1 (1)	1 (1)	>0.999
Mut. *TP53*	0	1 (1)	1 (1)	>0.999
**High-Dose Chemotherapy and Autologous Stem Cell Transplantation**				
Conditioning regimen—no. (%)				
Busulfan/Cyclophosphamide	25 (80)	63 (80)	88 (80)	>0.999
Busulfan/Melphalan	4 (13)	9 (11)	13 (12)	>0.999
Treosulfan/Melphalan	2 (7)	7 (9)	9 (8)	>0.999
Median time to ASCT (IQR)—months	3.4 (3.2–3.8)	3.7 (3.2–4.5)	3.7 (3.2–4.3)	0.153
Median CD34+ transfused—10^6^/kg BW	3.81 (3.1–5.2)	4.20 (3.3–5.2)	4.17 (3.2–5.2)	0.448

**Table 2:** Outcome of patients according to DTA-CH (clonal hematopoiesis) status after autologous stem cell transplantation (ASCT). CR: complete remission.

Parameter	CH (*n* = 31)	No CH (*n* = 79)	*p*
**Survival**			
Median follow up for OS—months	93.0	46.1	-
Median progression free survival—months	16.7	26.9	0.287
Median overall survival—months	54.4	80.9	0.440
2-year progression free survival probability (95% CI)	0.39 (0.34–0.57)	0.46 (0.22–0.56)	0.391
2-year overall survival probability (95%CI)	0.54 (0.35–0.71)	0.63 (0.49–0.73)	0.251
30-day mortality—no. (%)	0	0	>0.999
Allogeneic transplantation in CR2—no. (%)	6 (19)	19 (24)	0.801

## Data Availability

The data generated during and/or analyzed during this study are available from the corresponding author on reasonable request.

## Supplementary Materials

The following are available online at https://www.mdpi.com/article/10.3390/cancers13133190/s1, Figure S1: Distribution of mutations and variant allele frequencies (VAFs); Figure S2: Survival according to subgroups of clonal hematopoiesis (CH); Figure S3: Survival according to different VAF (variant allele frequencies) cutoffs.
