# Supplementary material for: Clonal Hematopoiesis after Autologous Stem Cell Transplantation Does Not Confer Adverse Prognosis in Patients with AML
Source: Cancers (Basel). 2021 Jun 25;13(13):3190. doi: 10.3390/cancers13133190 (PMC8267699; doi:10.3390/cancers13133190)

Clonal hematopoiesis after autologous stem cell transplantation does not confer adverse prognosis in patients with AML following autologous stem cell transplantation

Supplemental Material

Figure S1: Distribution of mutations and variant allele frequencies (VAFs). A: Absolute numbers of CH-mutations in *DNMT3A*, *TET2* and *ASXL1* detected at diagnosis and during complete remission in the absence of leukemia-related mutations. **B:** Variant allele frequencies of CH-related mutations in *DNMT3A*, *TET2* and *ASXL1* observed during complete remission. Grey dashes indicate medians.

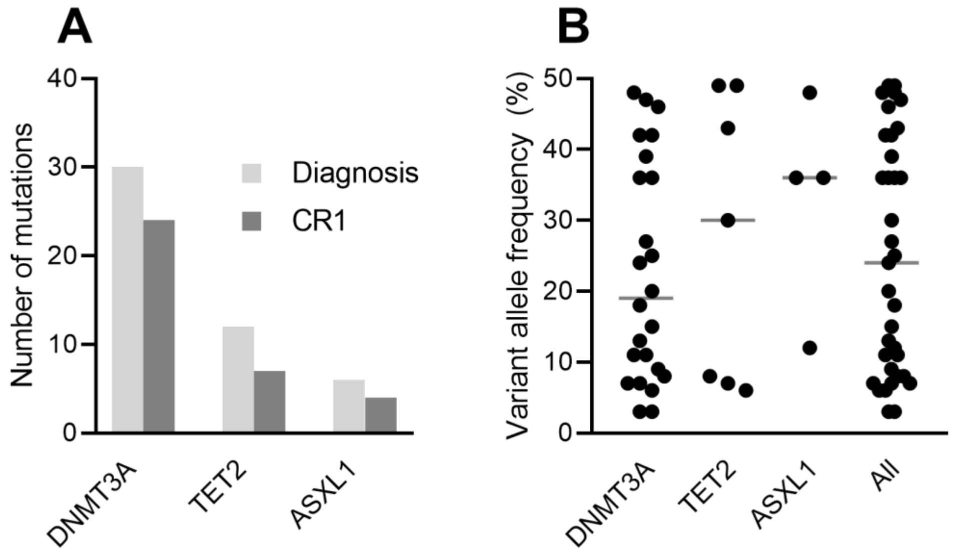

Figure S2: Survival according to subgroups of clonal hematopoiesis (CH). Kaplan-Meier survival estimates of patients with CH-related mutations in *DNMT3A*, *TET2* and *ASXL1* according to mutated gene. **A-B:** Progression-free (Panel A) and overall survival (Panel B) of patients with and without CH-related mutations in *DNMT3A.* **C-D:** Progression-free (Panel C) and overall survival (Panel D) of patients with and without CH-related mutations in *TET2*. **E-F:** Progression-free (Panel E) and overall survival (Panel F) of patients with and without CH-related mutations in *ASXL1.* **G-H:** Progression-free (Panel G) and overall survival (Panel H) of patients with and without CH-related mutations in *ASXL1* or *TET2*.

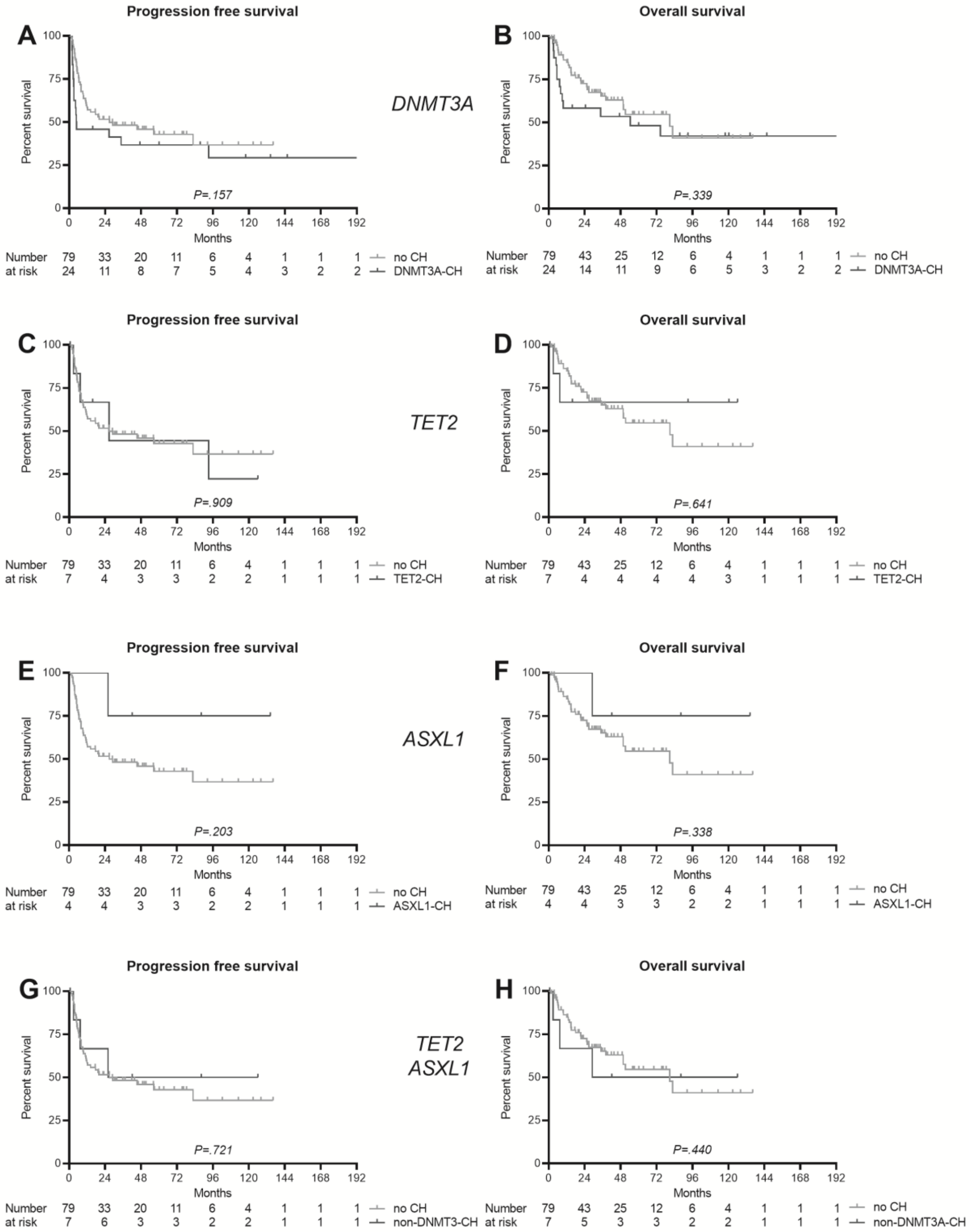

Figure S3: Survival according to different VAF (variant allele frequencies) cutoffs. Kaplan-Meier survival estimates of patients with and without CH determined at different VAF (variant allele frequency) cutoffs. **A-B:** Cutoff at 5% VAF. **C-D:** Cutoff at 10% VAF.

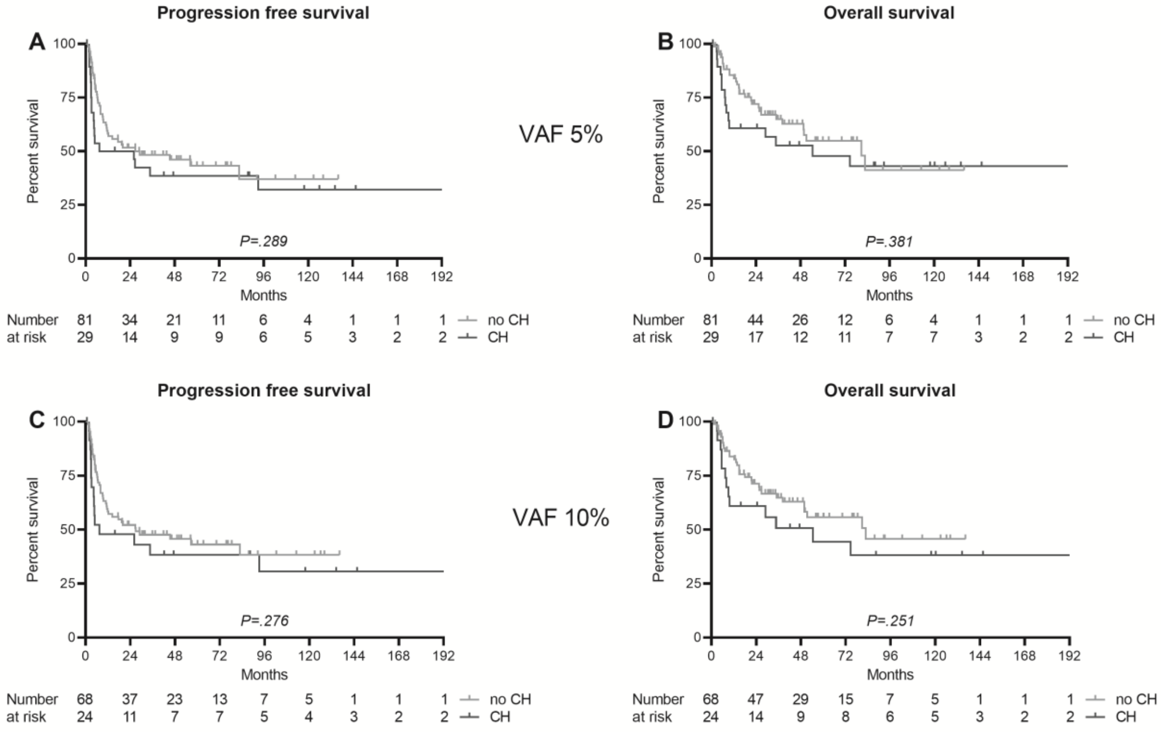

Supplement: Supplementary file 1 [file cancers-13-03190-s001.zip › cancers-1251461-supplementary.pdf]
